# 2-Amino-5-chloro­pyridinium *cis*-diaqua­dioxalatochromate(III) sesquihydrate

**DOI:** 10.1107/S1600536812023392

**Published:** 2012-05-26

**Authors:** Ichraf Chérif, Jawher Abdelhak, Mohamed Faouzi Zid, Ahmed Driss

**Affiliations:** a Laboratoire de Matériaux et Cristallochimie, Faculté des Sciences de Tunis, Université de Tunis El Manar, 2092 Manar II Tunis, Tunisia

## Abstract

In the crystal structure of the title compound, (C_5_H_6_ClN_2_)[Cr(C_2_O_4_)_2_(H_2_O)_2_]·1.5H_2_O, the Cr^III^ atom adopts a distorted octa­hedral geometry being coordinated by two O atoms of two *cis* water mol­ecules and four O atoms from two chelating oxalate dianions. The *cis*-diaqua­dioxalatochromate(III) anions, 2-amino-5-chloro­pyridinium cations and uncoordinated water mol­ecules are linked into a three-dimensional supra­molecular array by O—H⋯O and N—H⋯O hydrogen-bonding inter­actions. One of the two independent lattice water molecules is situated on a twofold rotation axis.

## Related literature

For structural characterization of salts containing the [Cr(C_2_O_4_)_2_(H_2_O)_2_]^−^ anion with various cations see: Bélombé *et al.* (2009 ▶); Nenwa *et al.* (2010 ▶); Chérif *et al.* (2011 ▶). For the building of hybrid supra­molecular networks, see: Zhang *et al.* (2000 ▶); Paraschiv *et al.* (2007 ▶). For discussion of hydrogen bonding, see: Blessing (1986 ▶); Brown (1976 ▶).
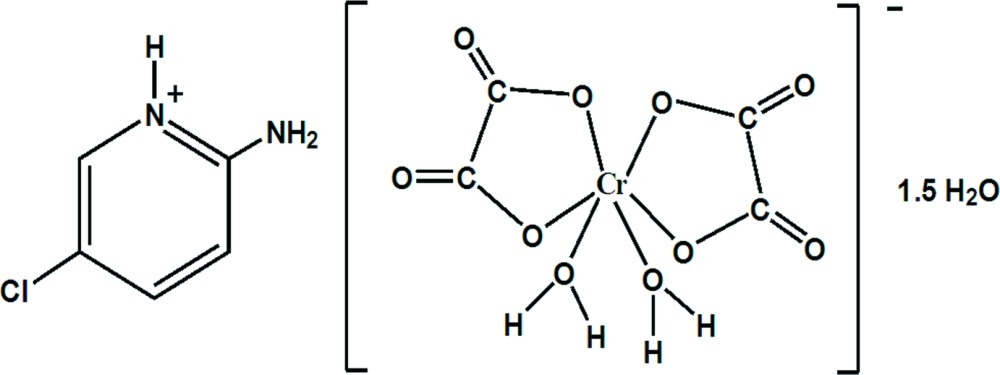


## Experimental

### 

#### Crystal data

(C_5_H_6_ClN_2_)[Cr(C_2_O_4_)_2_(H_2_O)_2_]·1.5H_2_O
*M* *_r_* = 420.66
Orthorhombic, 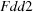

*a* = 11.376 (2) Å
*b* = 53.041 (3) Å
*c* = 10.413 (2) Å
*V* = 6283.1 (17) Å^3^
*Z* = 16
Mo *K*α radiation
μ = 0.96 mm^−1^
*T* = 298 K
0.42 × 0.32 × 0.13 mm

#### Data collection

Enraf–Nonius CAD-4 diffractometer
Absorption correction: ψ scan (North *et al.*, 1968 ▶) *T*_min_ = 0.792, *T*_max_ = 0.882
3854 measured reflections
3414 independent reflections
3180 reflections with *I* > 2σ(*I*)
*R*_int_ = 0.022
2 standard reflections every 120 min intensity decay: 5%

#### Refinement

*R*[*F* ^2^ > 2σ(*F* ^2^)] = 0.029
*wR*(*F* ^2^) = 0.074
*S* = 1.07
3414 reflections
243 parameters
8 restraints
H atoms treated by a mixture of independent and constrained refinement
Δρ_max_ = 0.30 e Å^−3^
Δρ_min_ = −0.31 e Å^−3^
Absolute structure: Flack (1983 ▶), 1608 Friedel pairs
Flack parameter: 0.000 (18)

### 

Data collection: *CAD-4 EXPRESS* (Duisenberg, 1992 ▶; Macíček & Yordanov, 1992 ▶); cell refinement: *CAD-4 EXPRESS*; data reduction: *XCAD4* (Harms & Wocadlo, 1995 ▶); program(s) used to solve structure: *SHELXS97* (Sheldrick, 2008 ▶); program(s) used to refine structure: *SHELXL97* (Sheldrick, 2008 ▶); molecular graphics: *DIAMOND* (Brandenburg, 1998 ▶); software used to prepare material for publication: *WinGX* (Farrugia, 1999 ▶).

## Supplementary Material

Crystal structure: contains datablock(s) I, global. DOI: 10.1107/S1600536812023392/kp2416sup1.cif

Structure factors: contains datablock(s) I. DOI: 10.1107/S1600536812023392/kp2416Isup2.hkl

Additional supplementary materials:  crystallographic information; 3D view; checkCIF report

## Figures and Tables

**Table 1:** Selected bond lengths (Å)

Cr—O1	1.9618 (19)
Cr—O2	1.9907 (19)
Cr—O5	1.9547 (19)
Cr—O6	1.9642 (18)
Cr—O1*W*	1.9978 (18)
Cr—O2*W*	1.9891 (19)

**Table 2:** Hydrogen-bond geometry (Å, °)

*D*—H⋯*A*	*D*—H	H⋯*A*	*D*⋯*A*	*D*—H⋯*A*
O1*W*—H11*W*⋯O4^i^	0.85 (2)	1.84 (3)	2.686 (3)	172 (3)
O1*W*—H12*W*⋯O3^ii^	0.85 (2)	1.94 (3)	2.769 (3)	163 (3)
O2*W*—H21*W*⋯O3^iii^	0.90 (2)	1.91 (2)	2.775 (3)	161 (3)
O2*W*—H21*W*⋯O4^iii^	0.90 (2)	2.37 (3)	2.909 (3)	118 (2)
O2*W*—H22*W*⋯O4*W*^iv^	0.89 (2)	1.89 (3)	2.770 (3)	176 (3)
O3*W*—H31*W*⋯O2^v^	0.88 (2)	2.12 (4)	2.979 (3)	167 (4)
O3*W*—H32*W*⋯O1	0.88 (2)	2.03 (4)	2.861 (3)	157 (4)
O4*W*—H4*W*⋯O6	0.90 (2)	2.12 (3)	3.011 (3)	173 (3)
N1—H1*A*⋯O8^vi^	0.86	2.15	2.911 (4)	147
N1—H1*B*⋯O3*W*^iv^	0.86	2.07	2.900 (4)	161
N2—H2⋯O8^vi^	0.86	2.13	2.900 (4)	150
N2—H2⋯O7^vi^	0.86	2.25	2.897 (4)	132

Symmetry codes: (i) 
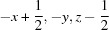
; (ii) 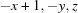; (iii) 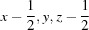; (iv) 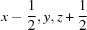; (v) 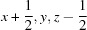; (vi) 
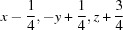
.

**Fig. 1.**
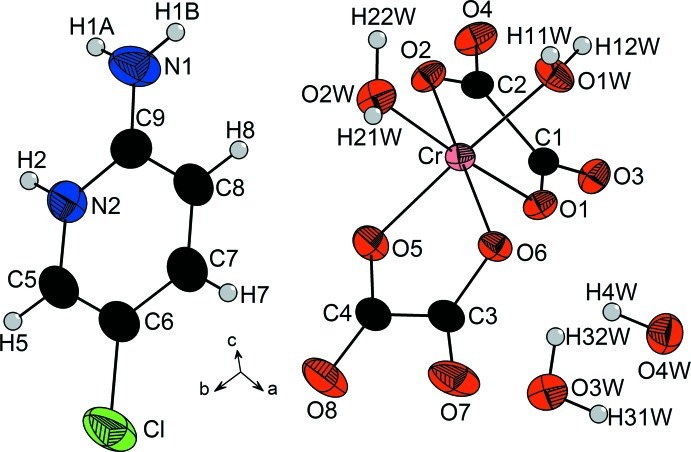
The asymmetric unit of (C5H6ClN2)[Cr(C2O4)2(H2O)2].1.5H2O with the atom-numbering scheme. Thermal ellipsoids are drawn at the 50% probability level for non-H atoms.

**Fig. 2.**
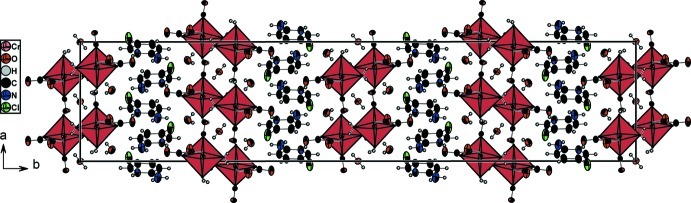
Projection of (C5H6ClN2)[Cr(C2O4)2(H2O)2].1.5H2O structure along the c axis.

**Fig. 3.**
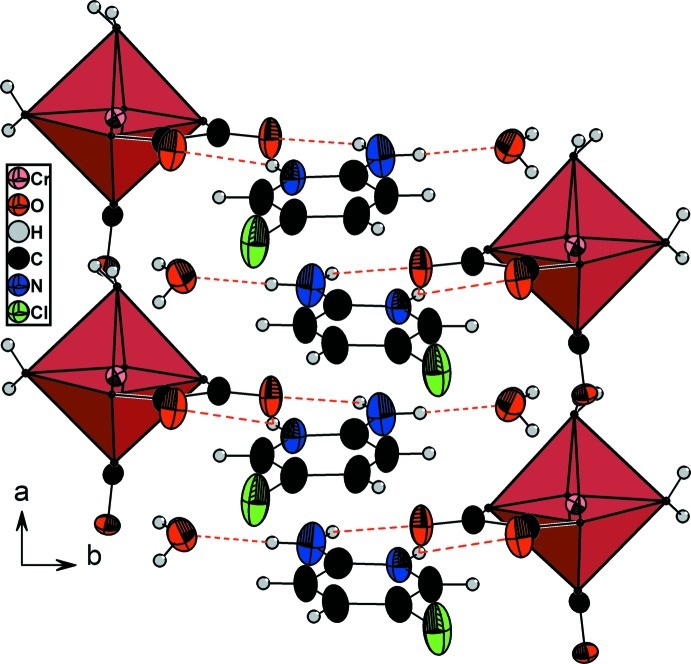
N—H···O hydrogen bonds (dashed lines) in (C5H6ClN2)[Cr(C2O4)2(H2O)2].1.5H2O showing the connection between positive and negative layers.

## supplementary crystallographic information

### Comment

It is well known that the use of hydrogen-bonding and π-π stacking interactions is a successful way to obtain a large variety of hybrid (organic/inorganic) compounds with extended supramolecular networks through self-assembly (Zhang *et al.*, 2000; Paraschiv *et al.*, 2007). Following this strategy, we recently published the structure of an organic-inorganic hybrid salt: 4-aminopyridinium *trans*-diaquadioxalatochromate(III) monohydrate (Chérif *et al.*, 2011). In this contribution, we report the crystal structure of an homologous salt with 2-amino-5-chloropyridinium as the organic cation.

The title compound appears to be the first member of salts of general formula (organic cation)[Cr(C_2_O_4_)_2_(H_2_O)_2_].xH_2_O where *x* = 0 or *x* = 1 in which the complex anion [Cr(C_2_O_4_)_2_(H_2_O)_2_]^-^ adopts the *cis* geometry. The asymmetric unit is formed by a [Cr(C_2_O_4_)_2_(H_2_O)_2_]^-^ anion, a (C_5_H_6_ClN_2_)^+^ cation and 1.5 water molecules [The O4W atom is located on a special position (1/2, 0, *z*)] (Fig. 1). In the complex anion, each chromium atom is six-coordinated in a distorted octahedral geometry with two O water molecules in *cis* position and four oxalato-O atoms from two chelating oxalate groups (Table 1). The four Cr—O(ox) distances range from 1.955 (2) to 1.991 (2) Å; three of them in the range 1.955 (2)–1.965 (2) Å are comparable to those reported in similar compounds (Bélombé *et al.*, 2009; Nenwa *et al.*, 2010; Chérif *et al.*, 2011) but the last one, Cr—O2, is slightly longer. The Cr—O(water) distances are shorter than those observed for the quinolinium and 4-dimethylaminopyridinium compounds (Bélombé *et al.*, 2009; Nenwa *et al.*, 2010).

The structure can be described as segregated positive (C_5_H_6_ClN_2_)^+^ and negative [[Cr(C_2_O_4_)_2_(H_2_O)_2_]^-^ + H_2_O] layers parallel to (010) (Fig. 2) and interconnected *via* N—H···O and O—H···O hydrogen bonds (Blessing, 1986; Brown, 1976). In fact, an extensive network of hydrogen bonds contributes to the stabilization of the structure. O—H···O hydrogen bonds involving all water molecules and some of the oxalato-O atoms provide the cohesion of the positive layers. The two N atoms of (C_5_H_6_ClN_2_)^+^ are hydrogen bonded to the peripheral O atoms of the oxalate groups (O8 and O7) and to the solvent water molecules (O3W) connecting the positive and negative layers (Fig. 3, Table 2).

### Experimental

Ethanol solutions of C_5_H_5_ClN_2_ (1 mmol) (10 mL) and H_2_C_2_O_4_.2H_2_O (2 mmol) (10 mL) were added to CrCl_3_.6H_2_O (1 mmol) dissolved in 10 mL of ethanol and stirred for 5 h. The resulting violet solution was left at room temperature and crystals suitable for X-ray diffraction were obtained after two weeks of slow evaporation.

### Refinement

All non hydrogen atoms were treated anisotropically. Water H atoms were initially located in a difference Fourier map and refined with restraints: d(O—H)=0.90 (2) Å and *U*_iso_(H)=1.5*U*_eq_(O). All other H atoms were constrained to an ideal geometry with d(C—H)=0.93 Å, d(N—H)=0.86 Å and *U*_iso_(H)=1.2*U*_eq_(C or N).

### Crystal data

**Table:**

(C_5_H_6_ClN_2_)[Cr(C_2_O_4_)_2_(H_2_O)_2_]·1.5H_2_O	*F*(000) = 3424
*M**_r_* = 420.66	*D*_x_ = 1.779 Mg m^−^^3^
Orthorhombic, *F**d**d*2	Mo *K*α radiation, λ = 0.71073 Å
Hall symbol: F 2 -2d	Cell parameters from 25 reflections
*a* = 11.376 (2) Å	θ = 10–15°
*b* = 53.041 (3) Å	µ = 0.96 mm^−^^1^
*c* = 10.413 (2) Å	*T* = 298 K
*V* = 6283.1 (17) Å^3^	Prism, violet
*Z* = 16	0.42 × 0.32 × 0.13 mm

### Data collection

**Table:**

Enraf–Nonius CAD-4 diffractometer	3180 reflections with *I* > 2σ(*I*)
Radiation source: fine-focus sealed tube	*R*_int_ = 0.022
Graphite monochromator	θ_max_ = 27.0°, θ_min_ = 2.7°
ω/2θ scans	*h* = −14→1
Absorption correction: ψ scan (North *et al.*, 1968)	*k* = −1→67
*T*_min_ = 0.792, *T*_max_ = 0.882	*l* = −13→13
3854 measured reflections	2 standard reflections every 120 min
3414 independent reflections	intensity decay: 5%

### Refinement

**Table:**

Refinement on *F*^2^	Secondary atom site location: difference Fourier map
Least-squares matrix: full	Hydrogen site location: inferred from neighbouring sites
*R*[*F*^2^ > 2σ(*F*^2^)] = 0.029	H atoms treated by a mixture of independent and constrained refinement
*wR*(*F*^2^) = 0.074	*w* = 1/[σ^2^(*F*_o_^2^) + (0.0316*P*)^2^ + 13.7688*P*] where *P* = (*F*_o_^2^ + 2*F*_c_^2^)/3
*S* = 1.07	(Δ/σ)_max_ < 0.001
3414 reflections	Δρ_max_ = 0.30 e Å^−^^3^
243 parameters	Δρ_min_ = −0.31 e Å^−^^3^
8 restraints	Absolute structure: Flack (1983), 1608 Friedel pairs
Primary atom site location: structure-invariant direct methods	Flack parameter: 0.000 (18)

### Special details

**Table:**

Geometry. All e.s.d.'s (except the e.s.d. in the dihedral angle between two l.s. planes) are estimated using the full covariance matrix. The cell e.s.d.'s are taken into account individually in the estimation of e.s.d.'s in distances, angles and torsion angles; correlations between e.s.d.'s in cell parameters are only used when they are defined by crystal symmetry. An approximate (isotropic) treatment of cell e.s.d.'s is used for estimating e.s.d.'s involving l.s. planes.
Refinement. Refinement of *F*^2^ against ALL reflections. The weighted *R*-factor *wR* and goodness of fit *S* are based on *F*^2^, conventional *R*-factors *R* are based on *F*, with *F* set to zero for negative *F*^2^. The threshold expression of *F*^2^ > σ(*F*^2^) is used only for calculating *R*-factors(gt) *etc*. and is not relevant to the choice of reflections for refinement. *R*-factors based on *F*^2^ are statistically about twice as large as those based on *F*, and *R*- factors based on ALL data will be even larger.

### Fractional atomic coordinates and isotropic or equivalent isotropic displacement parameters (Å^2^)

**Table:**

	*x*	*y*	*z*	*U*_iso_*/*U*_eq_	
Cr	0.28854 (3)	0.029597 (7)	0.12687 (3)	0.01808 (9)	
O1	0.45606 (16)	0.02882 (3)	0.17139 (17)	0.0219 (4)	
O2	0.27072 (17)	0.03331 (4)	0.31611 (17)	0.0238 (4)	
O3	0.57607 (15)	0.02584 (3)	0.34029 (18)	0.0245 (4)	
O4	0.37907 (17)	0.03041 (4)	0.49507 (17)	0.0295 (4)	
O5	0.29574 (17)	0.06593 (3)	0.09653 (16)	0.0286 (4)	
O6	0.32410 (18)	0.02782 (3)	−0.05749 (16)	0.0228 (4)	
O7	0.3518 (3)	0.05318 (4)	−0.2259 (2)	0.0467 (6)	
O8	0.3288 (3)	0.09378 (4)	−0.0606 (2)	0.0471 (6)	
O1W	0.28282 (17)	−0.00790 (3)	0.1438 (2)	0.0289 (4)	
H11W	0.228 (2)	−0.0153 (6)	0.103 (3)	0.043*	
H12W	0.313 (3)	−0.0148 (6)	0.210 (3)	0.043*	
O2W	0.11515 (17)	0.03017 (4)	0.10241 (18)	0.0297 (5)	
H21W	0.086 (3)	0.0282 (7)	0.023 (2)	0.045*	
H22W	0.075 (3)	0.0209 (6)	0.158 (3)	0.045*	
O3W	0.5956 (2)	0.05669 (5)	−0.0077 (3)	0.0541 (7)	
H31W	0.646 (3)	0.0476 (8)	−0.052 (4)	0.081*	
H32W	0.572 (4)	0.0474 (8)	0.058 (3)	0.081*	
O4W	0.5000	0.0000	−0.2206 (3)	0.0366 (7)	
H4W	0.448 (3)	0.0093 (7)	−0.177 (3)	0.055*	
C1	0.4779 (2)	0.02831 (4)	0.2921 (2)	0.0186 (5)	
C2	0.3677 (2)	0.03087 (4)	0.3782 (2)	0.0204 (5)	
C3	0.3332 (2)	0.04963 (5)	−0.1130 (3)	0.0264 (5)	
C4	0.3183 (3)	0.07223 (5)	−0.0199 (3)	0.0290 (6)	
C5	0.1933 (3)	0.15991 (6)	0.3233 (3)	0.0418 (8)	
H5	0.1933	0.1774	0.3230	0.050*	
C6	0.2315 (3)	0.14673 (6)	0.2190 (3)	0.0423 (7)	
C7	0.2297 (3)	0.12042 (6)	0.2198 (3)	0.0438 (8)	
H7	0.2549	0.1114	0.1482	0.053*	
C8	0.1909 (3)	0.10801 (6)	0.3254 (3)	0.0422 (8)	
H8	0.1899	0.0905	0.3262	0.051*	
C9	0.1518 (3)	0.12160 (6)	0.4344 (3)	0.0379 (7)	
N1	0.1138 (3)	0.11041 (6)	0.5402 (3)	0.0565 (9)	
H1A	0.0913	0.1193	0.6047	0.068*	
H1B	0.1115	0.0942	0.5445	0.068*	
N2	0.1552 (3)	0.14686 (5)	0.4280 (2)	0.0394 (6)	
H2	0.1319	0.1553	0.4938	0.047*	
Cl	0.27889 (13)	0.162724 (19)	0.08436 (9)	0.0737 (4)	

### Atomic displacement parameters (Å^2^)

**Table:**

	*U*^11^	*U*^22^	*U*^33^	*U*^12^	*U*^13^	*U*^23^
Cr	0.02037 (17)	0.02121 (17)	0.01267 (16)	−0.00028 (16)	−0.00167 (15)	0.00091 (14)
O1	0.0205 (9)	0.0299 (10)	0.0153 (8)	−0.0020 (7)	−0.0002 (7)	−0.0005 (7)
O2	0.0199 (9)	0.0364 (10)	0.0152 (8)	0.0007 (7)	0.0009 (7)	−0.0006 (7)
O3	0.0202 (9)	0.0318 (10)	0.0217 (9)	0.0023 (7)	−0.0022 (7)	−0.0021 (7)
O4	0.0292 (10)	0.0449 (12)	0.0144 (9)	−0.0055 (8)	−0.0002 (8)	−0.0022 (7)
O5	0.0426 (11)	0.0237 (9)	0.0195 (10)	0.0004 (8)	0.0002 (8)	−0.0017 (7)
O6	0.0323 (10)	0.0215 (9)	0.0147 (9)	−0.0017 (7)	0.0003 (8)	−0.0004 (6)
O7	0.0840 (19)	0.0369 (12)	0.0193 (10)	−0.0082 (12)	0.0086 (11)	0.0046 (9)
O8	0.0819 (18)	0.0244 (11)	0.0352 (12)	−0.0008 (11)	0.0065 (12)	0.0055 (9)
O1W	0.0335 (10)	0.0240 (9)	0.0290 (11)	−0.0057 (8)	−0.0117 (8)	0.0064 (8)
O2W	0.0230 (10)	0.0415 (12)	0.0247 (12)	−0.0020 (8)	−0.0070 (8)	0.0010 (8)
O3W	0.0566 (16)	0.0432 (14)	0.0626 (17)	0.0086 (12)	0.0283 (14)	0.0134 (12)
O4W	0.0340 (16)	0.0394 (18)	0.0365 (16)	0.0055 (13)	0.000	0.000
C1	0.0209 (12)	0.0191 (11)	0.0160 (11)	−0.0019 (9)	0.0000 (10)	−0.0007 (9)
C2	0.0211 (11)	0.0225 (11)	0.0177 (11)	−0.0028 (9)	0.0009 (10)	0.0008 (10)
C3	0.0349 (13)	0.0254 (12)	0.0189 (12)	−0.0019 (10)	−0.0013 (11)	0.0022 (10)
C4	0.0372 (14)	0.0234 (13)	0.0263 (13)	−0.0009 (11)	0.0028 (11)	0.0011 (11)
C5	0.063 (2)	0.0263 (15)	0.0361 (16)	0.0034 (14)	0.0054 (16)	−0.0047 (12)
C6	0.062 (2)	0.0331 (16)	0.0318 (15)	0.0050 (15)	0.0083 (16)	−0.0007 (13)
C7	0.069 (2)	0.0321 (15)	0.0305 (15)	0.0067 (15)	0.0075 (16)	−0.0089 (13)
C8	0.064 (2)	0.0254 (14)	0.0373 (16)	0.0024 (15)	0.0092 (16)	−0.0069 (12)
C9	0.0452 (19)	0.0375 (16)	0.0311 (15)	−0.0007 (14)	0.0043 (13)	−0.0044 (13)
N1	0.092 (3)	0.0399 (16)	0.0380 (16)	−0.0064 (16)	0.0192 (17)	−0.0017 (13)
N2	0.0595 (18)	0.0302 (13)	0.0284 (12)	0.0027 (12)	0.0068 (12)	−0.0092 (11)
Cl	0.1375 (11)	0.0385 (5)	0.0451 (5)	0.0074 (6)	0.0346 (7)	0.0065 (4)

### Geometric parameters (Å, º)

**Table:**

Cr—O1	1.9618 (19)	O3W—H32W	0.883 (19)
Cr—O2	1.9907 (19)	O4W—H4W	0.897 (18)
Cr—O5	1.9547 (19)	C1—C2	1.547 (3)
Cr—O6	1.9642 (18)	C3—C4	1.551 (4)
Cr—O1W	1.9978 (18)	C5—N2	1.363 (4)
Cr—O2W	1.9891 (19)	C5—C6	1.363 (4)
O1—C1	1.282 (3)	C5—H5	0.9300
O2—C2	1.286 (3)	C6—C7	1.396 (4)
O3—C1	1.231 (3)	C6—Cl	1.725 (3)
O4—C2	1.224 (3)	C7—C8	1.355 (5)
O5—C4	1.284 (3)	C7—H7	0.9300
O6—C3	1.297 (3)	C8—C9	1.417 (4)
O7—C3	1.209 (3)	C8—H8	0.9300
O8—C4	1.225 (3)	C9—N1	1.324 (4)
O1W—H11W	0.850 (18)	C9—N2	1.342 (4)
O1W—H12W	0.850 (18)	N1—H1A	0.8600
O2W—H21W	0.896 (18)	N1—H1B	0.8600
O2W—H22W	0.885 (18)	N2—H2	0.8600
O3W—H31W	0.880 (19)		
			
O5—Cr—O1	91.04 (8)	O4—C2—C1	119.3 (2)
O5—Cr—O6	83.15 (7)	O2—C2—C1	114.4 (2)
O1—Cr—O6	91.71 (8)	O7—C3—O6	125.9 (3)
O5—Cr—O2W	90.33 (9)	O7—C3—C4	120.4 (2)
O1—Cr—O2W	173.68 (8)	O6—C3—C4	113.7 (2)
O6—Cr—O2W	94.58 (8)	O8—C4—O5	126.1 (3)
O5—Cr—O2	93.82 (8)	O8—C4—C3	119.7 (2)
O1—Cr—O2	82.36 (7)	O5—C4—C3	114.3 (2)
O6—Cr—O2	173.31 (9)	N2—C5—C6	118.6 (3)
O2W—Cr—O2	91.39 (8)	N2—C5—H5	120.7
O5—Cr—O1W	175.72 (9)	C6—C5—H5	120.7
O1—Cr—O1W	89.42 (8)	C5—C6—C7	120.2 (3)
O6—Cr—O1W	92.58 (8)	C5—C6—Cl	119.7 (3)
O2W—Cr—O1W	89.67 (9)	C7—C6—Cl	120.1 (3)
O2—Cr—O1W	90.46 (8)	C8—C7—C6	119.7 (3)
C1—O1—Cr	114.86 (16)	C8—C7—H7	120.2
C2—O2—Cr	113.60 (17)	C6—C7—H7	120.2
C4—O5—Cr	114.69 (16)	C7—C8—C9	120.3 (3)
C3—O6—Cr	114.13 (16)	C7—C8—H8	119.8
Cr—O1W—H11W	116 (2)	C9—C8—H8	119.8
Cr—O1W—H12W	119 (3)	N1—C9—N2	119.9 (3)
H11W—O1W—H12W	120 (3)	N1—C9—C8	122.8 (3)
Cr—O2W—H21W	119 (2)	N2—C9—C8	117.3 (3)
Cr—O2W—H22W	115 (2)	C9—N1—H1A	120.0
H21W—O2W—H22W	110 (3)	C9—N1—H1B	120.0
H31W—O3W—H32W	107 (5)	H1A—N1—H1B	120.0
O3—C1—O1	125.3 (2)	C9—N2—C5	123.8 (3)
O3—C1—C2	120.5 (2)	C9—N2—H2	118.1
O1—C1—C2	114.2 (2)	C5—N2—H2	118.1
O4—C2—O2	126.3 (2)		

### Hydrogen-bond geometry (Å, º)

**Table:**

*D*—H···*A*	*D*—H	H···*A*	*D*···*A*	*D*—H···*A*
O1*W*—H11*W*···O4^i^	0.85 (2)	1.84 (3)	2.686 (3)	172 (3)
O1*W*—H12*W*···O3^ii^	0.85 (2)	1.94 (3)	2.769 (3)	163 (3)
O2*W*—H21*W*···O3^iii^	0.90 (2)	1.91 (2)	2.775 (3)	161 (3)
O2*W*—H21*W*···O4^iii^	0.90 (2)	2.37 (3)	2.909 (3)	118 (2)
O2*W*—H22*W*···O4*W*^iv^	0.89 (2)	1.89 (3)	2.770 (3)	176 (3)
O3*W*—H31*W*···O2^v^	0.88 (2)	2.12 (4)	2.979 (3)	167 (4)
O3*W*—H32*W*···O1	0.88 (2)	2.03 (4)	2.861 (3)	157 (4)
O4*W*—H4*W*···O6	0.90 (2)	2.12 (3)	3.011 (3)	173 (3)
N1—H1*A*···O8^vi^	0.86	2.15	2.911 (4)	147
N1—H1*B*···O3*W*^iv^	0.86	2.07	2.900 (4)	161
N2—H2···O8^vi^	0.86	2.13	2.900 (4)	150
N2—H2···O7^vi^	0.86	2.25	2.897 (4)	132

Symmetry codes: (i) −*x*+1/2, −*y*, *z*−1/2; (ii) −*x*+1, −*y*, *z*; (iii) *x*−1/2, *y*, *z*−1/2; (iv) *x*−1/2, *y*, *z*+1/2; (v) *x*+1/2, *y*, *z*−1/2; (vi) *x*−1/4, −*y*+1/4, *z*+3/4.

## References

[bb1] Bélombé, M. M., Nenwa, J. & Emmerling, F. (2009). *Z. Kristallogr.* **224**, 239–240.

[bb2] Blessing, R. H. (1986). *Acta Cryst.* B**42**, 613–621.

[bb3] Brandenburg, K. (1998). *DIAMOND* University of Bonn, Germany.

[bb4] Brown, I. D. (1976). *Acta Cryst.* A**32**, 24–31.

[bb5] Chérif, I., Abdelhak, J., Zid, M. F. & Driss, A. (2011). *Acta Cryst.* E**67**, m1648–m1649. 10.1107/S1600536811044837 PMC3238586 22199477

[bb6] Duisenberg, A. J. M. (1992). *J. Appl. Cryst.* **25**, 92–96.

[bb7] Farrugia, L. J. (1999). *J. Appl. Cryst.* **32**, 837–838.

[bb8] Flack, H. D. (1983). *Acta Cryst.* A**39**, 876–881.

[bb9] Harms, K. & Wocadlo, S. (1995). *XCAD4* University of Marburg, Germany.

[bb10] Macíček, J. & Yordanov, A. (1992). *J. Appl. Cryst.* **25**, 73–80.

[bb11] Nenwa, J., Belombe, M. M., Ngoune, J. & Fokwa, B. P. T. (2010). *Acta Cryst.* E**66**, m1410. 10.1107/S1600536810040353 PMC3009100 21588840

[bb12] North, A. C. T., Phillips, D. C. & Mathews, F. S. (1968). *Acta Cryst.* A**24**, 351–359.

[bb13] Paraschiv, C., Ferlay, S., Hosseini, M. W., Kyritsakas, N., Planeix, J. M. & Andruh, M. (2007). *Rev. Roum. Chim.* **52**, 101–104.

[bb14] Sheldrick, G. M. (2008). *Acta Cryst.* A**64**, 112–122. 10.1107/S0108767307043930 18156677

[bb15] Zhang, L., Cheng, P., Tang, L. F., Weng, L. H., Jiang, Z. H., Liao, D. Z., Yan, S. P. & Wang, G. L. (2000). *Chem. Commun.* pp. 717–718.

